# Epitope analysis and protection by a ROP19 DNA vaccine against *Toxoplasma gondii*

**DOI:** 10.1051/parasite/2016017

**Published:** 2016-04-07

**Authors:** Jian Zhou, Lin Wang, Gang Lu, Aihua Zhou, Meiyan Zhu, Qihang Li, Zhilin Wang, Miradel Arken, Ao Wang, Shenyi He

**Affiliations:** 1 Department of Parasitology, Shandong University School of Medicine250012 Jinan Shandong Province PR China; 2 Department of Ji Nan Children’s Hospital 250022 Jinan Shandong Province PR China; 3 Department of Pediatrics, Provincial Hospital Affiliated to Shandong University, Shandong University School of Medicine 250021 Jinan Shandong Province PR China

**Keywords:** DNA vaccine, Immunity, ROP19, *Toxoplasma gondii*, BALB/c mice

## Abstract

We used bioinformatics approaches to identify B-cell and T-cell epitopes on the ROP19 protein of *Toxoplasma gondii*. Then, we constructed plasmids with ROP19 (pEGFP-C1-ROP19) and injected them into BALB/c mice to test the immunoprotection induced by this vaccine candidate. The results showed that immunization with pEGFP-C1-ROP19 induced effective cellular and humoral immune responses in mice; specifically, high serum levels of *T. gondii*-specific IgG and increased interferon-gamma production by splenocytes. Furthermore, the mice vaccinated with pROP19 had significantly fewer brain cysts (583 ± 160) than the mice injected with phosphate-buffered saline (1350 ± 243) or with the control plasmid, pEGFP-C1 (1300 ± 167). Compared with PBS-treated mice, those immunized with pROP19 had only 43% of the number of brain cysts. These results suggest that the DNA vaccine encoding ROP19 induced a significant immune response and provided protection against a challenge with *T. gondii* strain PRU cysts.

## Abstract (fr)

Nous avons utilisé des approches bio-informatiques pour identifier des épitopes de lymphocytes B et de lymphocytes T sur la protéine ROP19 de *Toxoplasma gondii*. Ensuite, nous avons construit des plasmides avec ROP19 (pEGFP-C1-ROP19) et les avons injectés à des souris BALB/c pour tester l’immunoprotection induite par ce candidat vaccin. Les résultats ont montré que l’immunisation avec pEGFP-C1-ROP19 a induit des réponses immunitaires cellulaires et humorales efficaces chez la souris, en particulier des taux sériques élevés d’IgG spécifiques à *T. gondii* et une production accrue d’interféron gamma par les splénocytes. En outre, les souris vaccinées avec pROP19 avaient significativement moins de kystes cérébraux (583 ± 160) que les souris injectées avec du tampon phosphate salin (1350 ± 243) ou avec le plasmide témoin pEGFP-C1 (1300 ± 167). En comparaison avec les souris traitées au PBS, celles qui sont immunisées avec pROP19 avaient seulement 43 % du nombre de kystes dans le cerveau. Ces résultats suggèrent que le vaccin à ADN codant pour la ROP19 induit une réponse immunitaire significative et fournit une protection contre une provocation avec des kystes de la souche PRU de *T. gondii*.

## Introduction

Toxoplasmosis caused by *Toxoplasma gondii* is an important zoonotic disease. Worldwide, one third of the population is affected by *T. gondii* to varying degrees [18]. *Toxoplasma gondii* can produce extensive and fatal tissue damage [16]. The invasion and cleaving of actively dividing tachyzoites is directly responsible for toxoplasmosis [3, 9]. According to epidemiological surveys, there is a wide distribution and a high prevalence of *T. gondii* in many areas of the world [14, 22]. Several studies have shown that DNA vaccines might be an effective means of protection against toxoplasmosis because these vaccines elicit long-lasting humoral and cell-mediated immunity and provide protection against parasitic infections [1, 15]. However, previous reports investigating the immunoprotection induced by DNA vaccines have largely focused on the tachyzoites of a virulent strain (RH strain) [14, 23, 28]. The bradyzoite is an important stage of *T. gondii* that infects mammals, so developing gene vaccines against the bradyzoite is urgently needed.

As an interdisciplinary science, bioinformatics has been widely used to predict protein structures, functions, and epitopes [19]. Prediction of epitopes plays a significant role in the immunogenicity design of new vaccines [2, 13]. Additionally, DNA vaccines are a viable strategy for preventing toxoplasmosis, and ROP family genes encoding *T. gondii* proteins are excellent candidates for use in designing new DNA vaccines. Previous studies indicated that ROP1 was a potent stimulator of humoral and cellular immune responses. Similarly, ROP16 has been used in a promising vaccine candidate against toxoplasmosis. Another study found that a ROP18 vaccine construct could enhance the amount of *T. gondii*-specific cytotoxic T lymphocytes, Th1 responses, and survival time, which suggested that ROP18 is a promising vaccine candidate against infection with *T. gondii.* In addition, ROP38 showed high protective effects against chronic toxoplasmosis [4, 24–26].

ROP19, a member of the ROP family, plays an important role in the parasitophorous vacuolar membrane and is a kind of active kinase located in the rhoptries and parasitophorous vacuole [17]. To our knowledge, there have not been any evaluations based on the B-cell epitope index of the immune efficacy of *T. gondii* ROP19 as a target for DNA antigenicity generation. In this study, we analyzed the antigenic characteristics of ROP19 using bioinformatics analyses. We constructed a DNA vaccine expressing the ROP19 antigen (pROP19) and then examined its expression in eukaryotic cells. The immunogenicity and protective efficacy of this DNA vaccine were investigated by determining the *T. gondii*-specific IgG levels and counting the number of brain cysts following infection.

## Materials and methods

### Prediction of T-cell epitopes and linear B-cell epitopes

Because *T. gondii* is an intracellular parasite, T-cell-mediated cellular immunity plays an important role in *T. gondii* infection [7]. The Immune Epitope Database (IEDB) (http://tools.immuneepitope.org/mhcii) online service was used to analyze the half maximal inhibitory concentration (IC_50_) values of peptides that bind to the major histocompatibility complex (MHC) class II molecules that recognize ROP19 and SAG1. The linear B-cell epitopes of ROP19 were analyzed by DNASTAR software, and the peptides that had a good antigenic index and surface probability were shown.

### *T. gondii* parasites and mice

Six- to eight-week-old female BALB/c mice were obtained from the Shandong University Laboratory Animal Center. All mice had free access to food and tap water, and all procedures used in this study were approved by the Ethics Committee on Animal Experiments of the Medical School of Shandong University.

*Toxoplasma gondii* PRU strain, a low virulence strain, was maintained in our laboratory by the passage of cysts in BALB/c mice, and the *T. gondii* (PRU strain) tachyzoites obtained from HFF cells were used to create soluble tachyzoite antigens (STAg) after being washed by centrifugation and resuspended in sterile phosphate-buffered saline (PBS). The parasite suspension was sonicated and centrifuged at 56 g for 20 min. The supernatant containing STAg was collected and kept at −70 °C until later use [27].

### Plasmid preparation and construction

The entire ROP19 open reading frame was amplified by PCR from the cDNA of *T. gondii* PRU strain tachyzoites with the following primers: forward, 5′-cggGGTACCATGAGAAGGCTGCTGCTTTC-3′ and reverse, 5′-cgGGATCCTCACTGAGATCTGGATGC-3′. The forward primer contains a *Kpn* I restriction site (underlined), while the reverse primer has a *Bam*H I restriction site (underlined). Trans Tag^TM^ High Fidelity DNA Polymerase (TransGen Biotech, Beijing, China) was used in the PCR amplification. The reaction was performed with the following optimized conditions: 1 cycle of 95 °C for 5 min, followed by 30 cycles of 95 °C for 30 s, 65 °C for 45 s, and 72 °C for 30 s. The final primer extension was for 10 min at 72 °C. PCR products were tested via electrophoresis on a 1.0% agarose gel.

The ROP19 PCR product was inserted into a pEASY-T1 vector (TransGen Biotech) to build a recombinant cloning plasmid. After sequencing, ROP19 was subcloned into a eukaryotic expression plasmid pEGFP-C1 (Novagen, USA) to obtain pROP19. Finally, the new recombinant plasmids were transfected into HEK-293T cells using Lipofectamine^TM^ 2000 reagent (Invitrogen, USA) according to the manufacturer’s instructions.

The Endotoxin-Free Mega Plasmid kit (Qiagen, Hilden, Germany) was used according to the manufacturer’s instructions to extract the plasmids. The A260/A280 measurement was used to detect the concentrations of pEGFP-C1 and pROP19. Sterile, endotoxin-free PBS was used to dilute the plasmids to a concentration of 1 mg/mL.

### Expression of ROP19 in HEK-293T cells

HEK-293T cells were maintained in a humidified 5% CO_2_ atmosphere at 37 °C in 6-well plates (Costar, USA). They were cultured in Dulbecco’s modified Eagle’s medium supplemented with streptomycin (100 mg/mL), penicillin (100 IU/mL), and 10% fetal bovine serum. When the density of HEK-293T cells reached 80–90%, the control vectors (pEGFP-C1) or the constructed eukaryotic expression plasmids (pROP19) were transfected into cells using Lipofectamine 2000 (Invitrogen, USA) according to the manufacturer’s instructions. Plasmids were mixed with Lipofectamine 2000 at a concentration of 10 μg/mL in Dulbecco’s modified Eagle’s medium (without antibiotics) and fetal bovine serum. The mixture was incubated at room temperature for 20 min before being added drop by drop into HEK-293T cells. The cells were incubated with the solutions for 6 h in a humidified 5% CO_2_ atmosphere at 37 °C. Finally, fresh cell culture medium was added, and the 6-well plates were returned to the cell incubator for 48 h. After incubation, the cells from the different groups (control, pEGFP-C1, and pROP19) were examined using a fluorescence microscope under a blue laser.

Western blot analyses of HEK-293T cells transfected with pROP19 were performed as follows: The cells were treated with RIPA Lysis Buffer containing 1 mM of the protease inhibitor phenylmethanesulfonyl fluoride and centrifuged at 13,000 × g for 10 min. Proteins were transferred onto a polyvinylidene fluoride membrane via electrophoresis at 60 V for 4 h using the Bio-Rad Transfer System (Bio-Rad, Hercules, CA). The membrane was saturated for 2 h with sealing fluid at room temperature and probed with goat anti-*T. gondii* ROP19 antibody diluted 1:10,000 in saturation buffer. The membrane was incubated for 2 h with a horseradish peroxide-labeled rabbit anti-goat IgG antibody (Sigma, USA) diluted 1:10,000 in saturation buffer, and signals were detected with a Super Sensitive Signal ECL (Enhanced Chemiluminescence) system.

### DNA immunization and experimental design

As shown in Table 1, three groups of mice (16 per group) were vaccinated three times by buttocks injection. Blood samples from all groups were collected at 2, 4, and 6 weeks after the initial vaccination and stored at −20 °C until their use in enzyme-linked immunosorbent assays (ELISAs) to assess serum IgG levels. To detect cytokine levels, 6 weeks after the first dose of vaccine, the splenocytes from four mice in each group were harvested under aseptic conditions, cultured, and cytokine levels in their supernatants were assessed using ELISAs. Two weeks after the final immunization, all the mice were infected intragastrically with 20 *T. gondii* PRU strain cysts. After a month of infection, the mice were sacrificed, the brain of each mouse was taken out and homogenized in 1 mL PBS. The number of cysts in each brain was detected by counting three 10 μL samples of the mixture, and the average numbers were used to evaluate the effect of the vaccine against *T. gondii.*

**Table 1.** The immunization process of BALB/c mice.

Group	Immunization times^a^		
	First time (μL)	Second time (μL)	Third time (μL)
PBS	100	100	100
pEGFP-C1	100	100	100
pEGFP-C1-ROP19	100	100	100

a All mice were injected three times at two-week intervals.

### Evaluation of *T. gondii*-specific IgG titers

We performed ELISAs to determine *T. gondii*-specific serum antibody levels. In short, the 96-well plates (Costar, USA) were coated with STAg (10 μg/well) and incubated at 4 °C overnight. The next day, plates were washed three times with ELISA solution and blocked with PBS containing 1% bovine serum albumin (BSA) for 1 h at 37 °C. Next, the plates were washed and incubated with the mouse sera diluted in PBS (1:100) for 1 h at 37 °C. After washing, plates were incubated with horseradish peroxidase-conjugated anti-mouse IgG (diluted 1:4,000 in PBS with 1% BSA) for 1 h at 37 °C. After washing, orthophenylenediamine (Sigma, USA) and 0.15% H_2_O_2_ were added to the plates. The plates were then incubated in the dark for 30 min at 37 °C, and the reaction was terminated by adding 2 M H_2_SO_4_. The OD was detected at 405 nm with an ELISA reader (ELX800, USA). All of the samples were run four times.

### Detection of cytokines

Spleens were isolated from four mice per group to detect the levels of cytokine production 6 weeks after the initial dose of vaccine. About 1 × 10^5^ spleen cells were stimulated with STAg (10 μg/mL each) in 96-well plates at 37 °C in 5% CO_2_. Cell-free supernatants were harvested to assay the interleukin (IL)-4 activity at 24 h, IL-10 activity at 72 h, and interferon-gamma (IFN-γ) activity at 96 h. The IL-4, IL-10, and IFN-γ concentrations were detected with commercial ELISA kits according to the manufacturer’s instructions (R&D Systems, USA). All samples were run four times.

### Statistical analyses

SPSS 17.0 was used to perform the statistical analysis. The significances of the differences in antibody levels and the number of brain cysts among the groups were determined with one-way analyses of variance (ANOVAs). When a significant difference (*p* < 0.05) was observed among treatments, Tukey’s studentized range test was used for posttest comparisons. The difference was considered statistically significant if *p* < 0.05.

### Ethics statement

This study was approved by the Institutional Animal Care and Use Committee of Shandong University under Contract 2011-0015. The animals were kept and the experiments were performed in accordance with the committee’s criteria for the care and use of laboratory animals. All mice were maintained in specific pathogen-free conditions, and all efforts were made to minimize suffering. Humane endpoints to reduce pain or distress in mice were used via euthanasia. Mice were sacrificed immediately using CO_2_ gas before the brains were removed. Generally, mice were placed in a chamber and CO_2_ was administered at a concentration of 60% to 70% over a 5-minute exposure time, after which the cervical dislocation method was sometimes used to ensure that effective euthanasia had occurred.

## Results

### Prediction of epitopes

**Figure 1.**
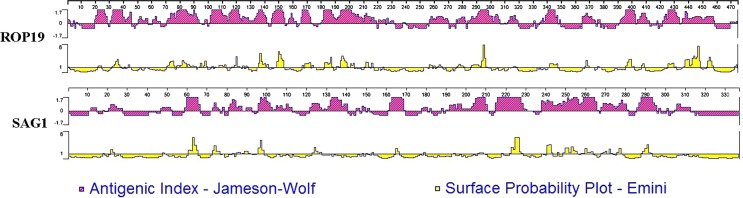
The linear-B-cell epitopes of ROP19 predicted by DNASTAR software in antigenic index and surface probability rules. The antigenic indexes are in red color; The surface probability plots are in yellow color.

The linear B-cell epitopes of SAG1 and ROP19 were predicted by DNASTAR software. Figure 1 shows the result of the ROP19 prediction. We found that ROP19 had a better antigenic index and surface probability than SAG1. Our data indicated that ROP19 had good linear B-cell epitopes compared to SAG1.

The online service IEDB was used to analyze T-cell epitopes of ROP19. The IC_50_ values of peptides binding to the MHC class II molecules of ROP19 were predicted. The T-cell epitopes on ROP19 identified by the bioinformatics analyses were predicted to have the ability to bind strongly to MHC class II molecules. Table 2 shows the minimum percentile ranks of each of the MHC II alleles on SAG1 and ROP19.

**Table 2.** IC_50_ values for ROP19 binding to MHC class II molecules obtained using the immune epitope database^a^.

MHC II Allele^b^	Start-Stop^c^		Percentile Rank^d^	
	SAG1	ROP19	SAG1	ROP19
HLA-DRB1*01:01	12–26	5–19	0.88	0.04
	35–49	160–174	2.74	0.04
H2-IAb	26–40	44–58	2.15	0.63
	297–313	536–550	2.81	2.19
H2-IAd	21–35	374–388	0.34	0.67
	168–182	539–553	1.22	0.18
H2-IEd	14–28	110–124	18.45	13.17
	34–48	520–534	30.62	13.22

a The immune epitope database (http://tools.immuneepitope.org/mhcii). The prediction was run three times.

b H2-IAb, H2-IAd, and H2-IEd alleles are mouse MHC class II molecules; the HLA-DRB1*01:01 allele is a human MHC class II molecule.

c We chose 15 amino acids for analysis each time.

d Low percentile rank = high level binding, high percentile rank = low level binding, IC_50_ values = percentile rank.

### Identification and expression of the recombinant plasmid

**Figure 2.**
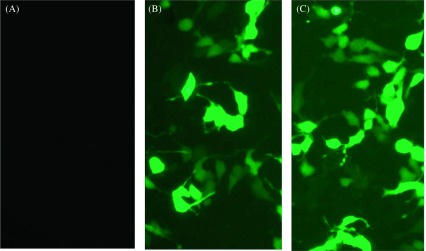
Direct immunofluorescence detection of the fusion protein in transfected HEK 293-T cells. (A) Untransfected cells were detected under blue light; (B) cells transfected with pEGFP-C1 were detected under blue light; (C) cells transfected with pROP19 were detected under blue light. Excitation wavelengths = 490 nm; Emission wavelengths = 520 nm.

HEK-293T cells transfected with pROP19 or the empty plasmid pEGFP-C1 were incubated for 48 h. Fluorescence microscopy was used to detect the green fluorescence of the protein in the cells. As shown in Figure 2, green fluorescence was detected in pROP19- and vector pEGFP-C1-transfected cells, while no fluorescence was observed in the untransfected cells (blank cells).

**Figure 3.**
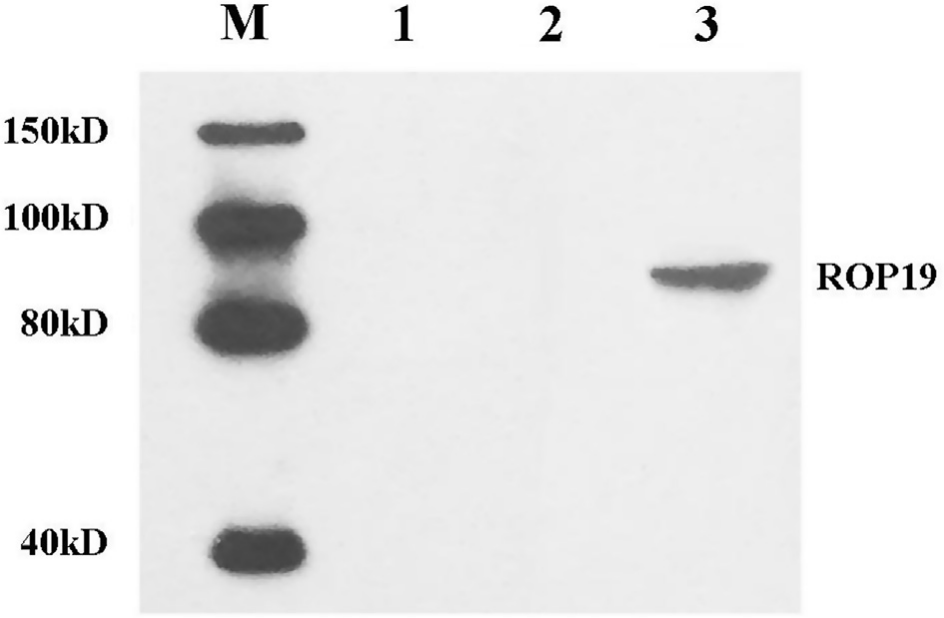
*In vitro* expression analysis of the constructs in HEK 293-T cells by Western blot. Protein marker (lane M), untransfected cells (lane 1), cells transfected with pEGFP-C1 (lane 2), and cells transfected with pROP19 (lane 3). The marker contains 150 kD, 100 kD, 80 kD, and 40 kD. The detected band is located between 80 kD and 100 kD.

The expression of the inserted gene was detected by Western blot. As shown in Figure 3, the expression of the ROP19 protein (compound protein of approximately 90 kDa) was detected in pROP19-transfected cells, while no protein band was observed for samples from the empty vector-transfected or blank cells.

### Antibody responses

**Figure 4.**
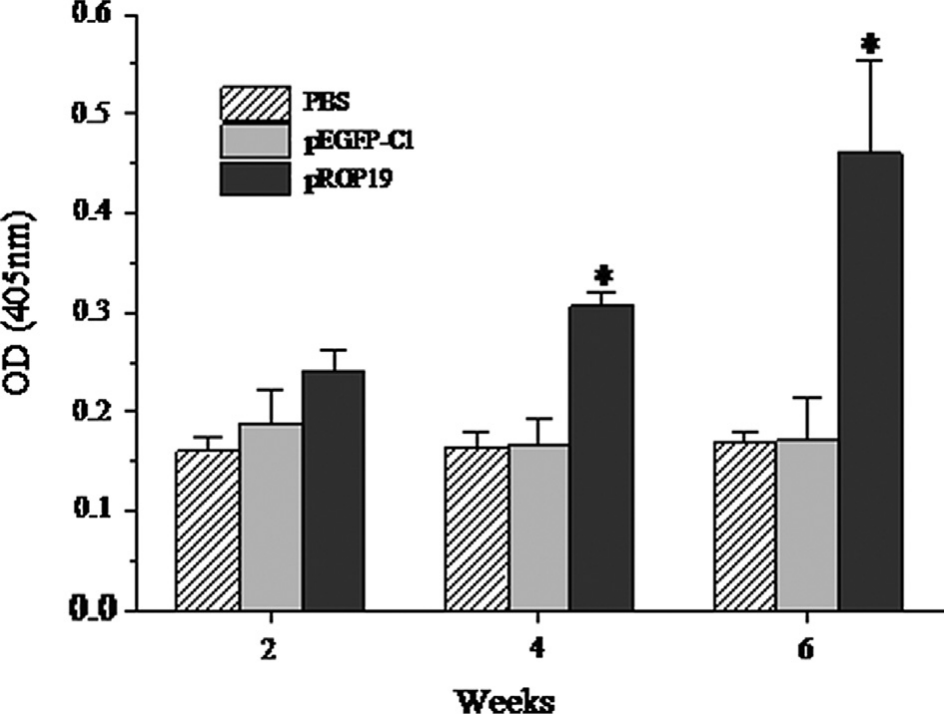
Measurement of specific IgG antibodies in sera of immunized mice. Sera were collected two days prior to each immunization and determined by ELISA. All samples were run four times. The results are the mean of three independent experiments and expressed as the mean of OD 405 ± SD. The statistical differences (*p* < 0.05) are indicated by *compared with PBS or pEGFP-C1.

ELISAs were used to detect the levels of *T. gondii-*specific IgG antibodies induced by these plasmids in mice at weeks 2, 4, and 6 after the first dose of vaccine. As shown in Figure 4, significantly higher levels of IgG antibodies were detected in the sera of mice immunized with pROP19 than those in the sera of mice from either of the control groups (*p* < 0.05). *T. gondii-*specific IgG antibodies were not detected in the mice injected with PBS or pEGFP-C1. Additionally, there was no statistical difference between the *T. gondii-*specific IgG antibodies in mice injected with PBS or pEGFP-C1. These results demonstrated that the constructed plasmid encoding *T. gondii* ROP19 protein induced a strong *T. gondii-*specific IgG antibody response in mice.

### Detection of cytokine production

To determine whether the DNA vaccine augmented cytokine responses, cultured supernatants of splenocytes taken from immunized mice 2 weeks after the last injection were assayed for the production of IFN-γ, IL-4, and IL-10. Table 3 shows that the level of IFN-γ in mice injected with pROP19 (485.04 ± 64.56) was higher than that in mice injected with PBS (54.44 ± 12.5) or pEGFP-C1 (54.30 ± 11.65) (*p* < 0.05). There was no statistical difference between the IFN-γ levels from the mice injected with PBS and those injected with pEGFP-C1. The highest level of IL-4 was detected in mice vaccinated with pROP19, but no statistically significant difference was found between these levels and those from mice vaccinated with PBS (*p* > 0.05). IL-10 was also detected in both the pROP19-vaccinated and control groups, but there were no significant differences in the IL-10 levels between any of the groups (*p* > 0.05).

**Table 3.** Cytokine production by splenocyte^a^ cultures from immunized BALB/c mice.

Group	Cytokine production (pg/mL)^b^		
	IFN-γ	IL-4	IL-10
PBS	54.44 ± 12.53	40.02 ± 9.18	39 ± 9.24
pEGFP-C1	54.30 ± 11.65	35.93 ± 8.95	41.73 ± 7.44
pEGFP-C1-ROP19	485.04 ± 64.559*	40.12 ± 6.29	41.33 ± 4.94

a Splenocytes from four mice per group two weeks after the final immunization.

b Values for IFN-γ at 96 h, IL-4 at 24 h, and IL-10 at 72 h are expressed as mean ± SD.

* Compared with PBS or pEGFP-C1 group, *p* < 0.05.

### Protection by pROP19 vaccine against PRU strain *T. gondii*

Two weeks after the last immunization, the vaccinated mice were intragastrically challenged with 20 PRU strain *T. gondii* cysts to evaluate the level of immunoprotection induced by the ROP19 DNA vaccine. Table 4 shows that significantly fewer brain cysts were observed in the mice immunized with pROP19 (583 ± 160) than in the mice injected with PBS (1350 ± 243) or pEGFP-C1 (1300 ± 167). Moreover, there was no difference in the number of brain cysts between the groups immunized with PBS and those immunized with pEGFP-C1 (*p* > 0.05).

**Table 4.** Cysts in injected mice after challenge by cysts of PRU strain.

Challenged group^a^	Brain cysts per mouse (mean ± SD)^b^
PBS	1350 ± 243
pEGFP-C1	1300 ± 167
pROP 19	583 ± 160*

a Ten mice from each group were challenged intragastrically by 20 cysts two weeks after the last immunization. All samples were run four times.

b The mean number of cysts of each group (four mice) was obtained from all mice brain cysts in the group.

* Compared with PBS or pEGFP-C1 group, *p* < 0.05.

## Discussion

In our study, the B-cell and T-cell epitopes of ROP19 were fully predicted by software and online services. DNASTAR software was used to analyze the sequence of ROP19 protein. As shown in Figure 1, its good antigenic index and surface probability indicated the presence of B-cell epitopes, which suggests that ROP19 has a strong potential to act as a beneficial B-cell antigen. We also used the online service IEDB to predict ROP19 T-cell epitopes and found several potential T-cell epitopes on the protein. As shown in Table 2, its low IC_50_ value indicates that ROP19 has viable T-cell epitopes.

IgG antibodies play a significant role in protecting mice from being infected with *T. gondii* and can prevent the parasite from attaching to the host cells [10]. In this study, the DNA vaccine pROP19 induced both humoral and cellular immune responses in the immunized mice. Compared with the control groups, a higher level of IgG antibodies was detected in mice injected with pROP19.

Additionally, we detected the levels of cytokine production (IFN-γ, IL-4, and IL-10) by spleen cells from the immunized mice to further characterize the polarization of the immune response. Each of the measured cytokines plays an important role in host resistance against *T. gondii*. The immunity mediated by the Th1 cytokine IFN-γ is important in controlling the replication of the protozoan [11, 12, 20]. IFN-γ also plays an important role in restricting the growth of *T. gondii* in the acute phase of the infection and in preventing the reactivation of parasites from dormant cysts [21]. Additionally, reports indicate that the Th2 cytokines (IL-4 and IL-10) generated during *T. gondii* infection are important in the immune response to this parasite. IL-4 is believed to prevent the host from succumbing to early Th1-polarized hyperactive immune responses, and the production of IFN-γ is impeded by high levels of IL-4 [5, 8].

Our results show that the humoral and cellular immune responses were successfully induced in mice vaccinated with pROP19. Additionally, the level of IFN-γ induced in the pROP19-vaccinated group was higher than that in the control groups. However, the production levels of IL-4 and IL-10 were similar in all three groups. Not surprisingly, the IFN-γ level in the pROP19-vaccinated mice was significantly higher than the levels of IL-4 and IL-10 in these mice. Our results indicate that the immune response stimulated by ROP19 was mainly a Th1 cell immune response.

Twenty PRU strain *T. gondii* cysts were used to challenge the immunized BALB/c mice to assess the efficiency of protection against *T. gondii*. The PRU strain of *T. gondii* is notably less virulent in mice than most other *T. gondii* strains. However, no vaccine has been reported to provide full protection against an intragastric challenge with cysts of any *T. gondii* strain. One study reported that the number of brain cysts was reduced by 77% in mice injected with three DNA plasmids (a combination of pVAX1-SAG2C, pVAX1-SAG2D, and pVAX1-SAG2X) compared with the amount of cysts in the control group mice [27]. Another study showed that in C57BL/6 mice and C3H/HeJ mice, their constructed vaccine (rROP2 + rROP4 + rSAG1) reduced the number of cysts by 90% and 71%, respectively, compared with the amounts of cysts in the control groups [6]. All the mice appear to be protected by a vaccine. In this study, a significant reduction in the number of brain cysts was found in BALB/c mice injected with pROP19 (57%). Although pROP19 showed lower efficacy compared with the multigene vaccines described above, it had higher efficacy than pSAG5A. Not surprisingly, pROP19 indicated more effective protection compared to other single gene vaccines, such as ROP38, infection with only 10 cysts of *T. gondii* PRU strain in the experiment, and SAG5A. [12, 24]. Therefore, ROP19 could be a candidate for a DNA vaccine against PRU strain *T. gondii*.

According to previous reports, many DNA vaccines, such as ROP16 and ROP18, were identified to be potential candidates against a virulent strain [16, 25]. However, most infections with toxoplasmosis are chronic in the form of cysts. As a result, construction of a DNA vaccine against a low virulence strain is significant. As shown in ToxoDB 10.0 (http://toxodb.org/toxo/) (Gene ID: TGME49_242240), ROP19 protein expression is upregulated in the low virulence strain, which prompted us to evaluate whether ROP19 could elicit effective immune responses against infection with a low virulence strain of *T. gondii* in the mouse model. Therefore, we chose ROP19 to evaluate the protective effect against chronic toxoplasmosis and obtained positive results.

In conclusion, we used bioinformatics approaches to identify B-cell epitopes and T-cell epitopes on *T. gondii* ROP19 protein. Then, the constructed plasmids containing the ROP19 gene were injected into BALB/c mice to test the immunoprotection induced by this vaccine candidate. Our data showed that an effective cellular and humoral immune response could be induced by vaccination with pROP19 in mice. Additionally, the number of cysts in the brains of the mice injected with pROP19 was reduced to 43% of the number of cysts in the brains of PBS-treated mice. This DNA vaccine was proven to be effective at inducing protection against infection by PRU strain *T. gondii* cysts.

## Conflict of interest

The authors declare that they have no competing interests.
